# ﻿Notes on the genus *Diestramima* Storozhenko, 1990 (Orthoptera, Rhaphidophoridae) with description of three new species from China

**DOI:** 10.3897/zookeys.1231.143063

**Published:** 2025-03-11

**Authors:** Qidi Zhu, Hao Xu, Ruigang Yang, Fuming Shi

**Affiliations:** 1 College of Life Sciences, Hebei University, Baoding 071002, China Hebei University Baoding China; 2 College of Agronomy, Jiangxi Agricultural University, Nanchang 330045, China Jiangxi Agricultural University Nanchang China; 3 Scientific Research Academy of Guangxi Environmental Protection, Nanning 530022, China Scientific Research Academy of Guangxi Environmental Protection Nanning China

**Keywords:** China, *Diestramima*, morphology, new species, taxonomy

## Abstract

Three new species of the genus *Diestramima* are described from China: *Diestramimaligula* Zhu & Shi, **sp. nov.**, *Diestramimalongzhouensis* Zhu & Shi, **sp. nov.**, and *Diestramimanapoensis* Zhu & Shi, **sp. nov.** Moreover, the females of *Diestramimaparabispinosa* Qin, Wang, Liu & Li, 2016 and *Diestramimadistincta* Gorochov, 2010 are described for the first time. Images illustrating the morphology of these species are provided.

## ﻿Introduction

Storozhenko (1990) established the genus *Diestramima* and assigned *Diestrammenapalpata* Rehn, 1906 as the type species. Subsequently, 34 species have been described in or transferred to the genus *Diestramima* based on morphological characters (Storozhenko 1990; Gorochov and Storozhenko 1992, 2015; Gorochov 1994, 1998, 2002, 2010; Liu and Zhang 2001; Storozhenko and Dawwrueng 2014; Qin et al. 2016; Zhu and Shi 2018; Wang et al. 2019; Cigliano et al. 2024). Gorochov and Storozhenko (2019) classified *Diestramima*into three subgenera, and Zhu et al. (2022) described six new species based on morphological and molecular evidence. Recently, Zhu et al.’s (2022) phylogenetic analysis did not support this three-subgenera classification system. He et al. (2023) reconstructed the phylogeny of *Diestramima* based on three mitochondrial genes and found that the topology was consistent with Zhu et al. (2022); He et al. (2023) published two new species and a junior synonym.

The genus *Diestramima* is mainly distributed across China, Vietnam, Laos, Myanmar, and the Indian Subcontinent. These insects prefer dark, moist habitats and are typically nocturnal, active at night, but hiding under leaves or loose bark during the day. Until now, the genus *Diestramima* has included 41 species, 31 species of which are recorded from China.

## ﻿Materials and methods

Specimens were collected by hand at night. The genitalia were dissected with an insect needle. Images were taken with a Zeiss AxioCam ICc5 digital camera attached to a Zeiss Stereo Discovery V12 microscope and edited with ADOBE PHOTOSHOP 2022. The measurements follow Zhu and Shi (2018). The type specimens of the new species are deposited in the Museum of Hebei University, Baoding, China (HBU).

## ﻿Results

**Genus *Diestramima* Storozhenko, 1990**

### ﻿ Diestramima ligula

Taxon classificationAnimaliaOrthopteraRhaphidophoridae

Zhu & Shi sp. nov.

8DD352D9-1E63-50B9-80A1-F5F203D45680

https://zoobank.org/69B04028-7993-490B-8338-6B6A27CB6EE7

Fig. 1

#### Material examined.

***Holotype***: China • ♂, Yunnan Province, Wenshan Zhuang and Miao Autonomous Prefecture, Maguan County, 22.8576°N, 104.0042°E, alt. 1790 m, 19.VI.2024, Hao Xu leg. ***Paratypes***: 1♂1♀, same data as for holotype.

**Figure 1.**
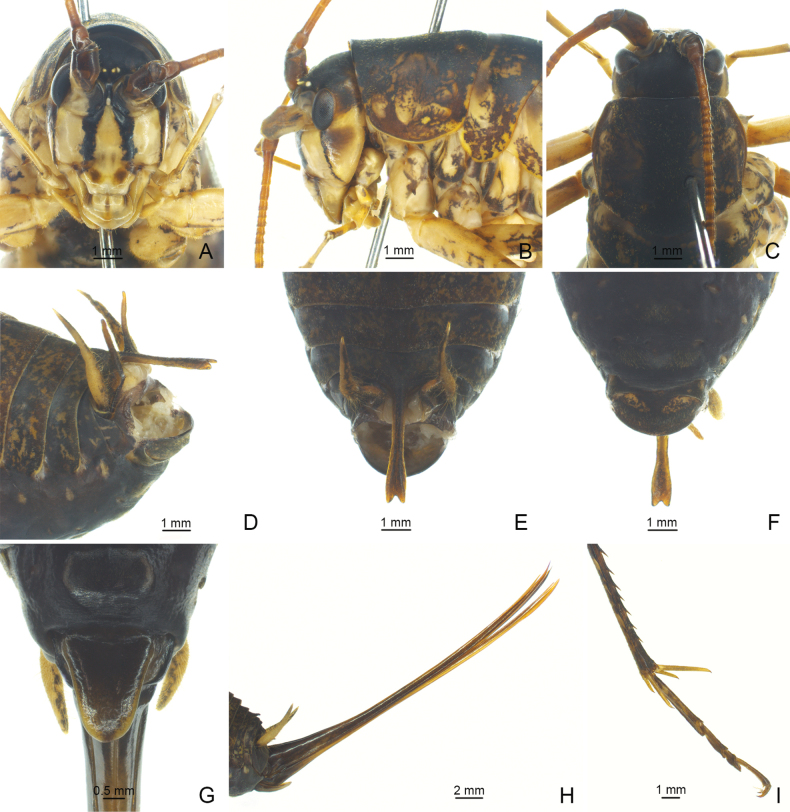
*Diestramimaligula* Zhu & Shi, sp. nov. **A–F, I** ♂ **A–C** head and pronotum **A** frontal view **B** lateral view **C** dorsal view **D–F** apex of abdomen **D** lateral view **E** dorsal view **F** ventral view **I** hind tarsus in lateral view **G, H** ♀ **G** subgenital plate **H** ovipositor in lateral view.

#### Diagnosis.

The new species is similar to *Diestramimamajor* Gorochov, 1998, but it can be easily distinguished from the latter by the shape of the seventh abdominal tergite and the paraproct of male. Posteromedian process of the seventh abdominal tergite of male rather long, surpassing apex of paraproct, basal half slender, paralleled on both sides, apical half slightly wider, apical area with a notch. Male paraproct digitiform, basal half broad, apical half slender, pointing upwards.

#### Description.

**Male.** Body medium-sized. Fastigium verticis with 2 conical tubercles, apices separated, obtusely rounded, pointing forwards. Eyes ovoid, protruding forwards; median ocellus oval, located between antennal sockets, lateral ocelli nearly circular, situated on lateral surface of basal rostral tubercles. Apical segment of maxillary palp obviously longer than subapical one, apex truncated. Pronotum broad, anterior margin of disc straight, posterior margin arcuate; lateral lobe longer than high, ventral margin arched. Mesonotum and metanotum short, posterior margin of mesonotum arcuate, posterior margin of metanotum straight. Fore coxa with 1 small spine; femur unarmed on ventral surface, internal genicular lobe with 1 small spine, external genicular lobe with 1 long spine; tibia with 2 inner spines and 2 outer spines on ventral surface, apex with 1 outer spine on dorsal surface and 1 pair of spines on ventral surface, between the paired ventral spines with 1 small spine. Middle femur unarmed on ventral surface, internal and external genicular lobes with 1 long spine respectively; tibia with 2 inner spines and 2 outer spines on ventral surface, apex with 1 pair of dorsal spines and 1 pair of ventral spines, between the paired ventral spines with 1 small spine. Hind femur with 12 inner spines on ventral surface, internal genicular lobe with 1 small spine, external genicular lobe unarmed; tibia with 22–25 inner spines and 26 or 27 outer spines on dorsal surface, subapex with 1 pair of dorsal spines, apex with 1 pair of dorsal spines and 2 pairs of ventral spines, intero-dorsal spine obviously shorter than hind basitarsus; hind basitarsus with 1 dorsal spine. Posterior margin of sixth abdominal tergite straight. Posteromedian process of seventh abdominal tergite rather long, surpassing apex of paraproct, basal half slender, paralleled on both sides, apical half slightly wider, apical area with a notch. Paraproct digitiform, basal half broad, apical half slender, pointing upwards. Cercus narrow, conical, apex acute. Genitalia with 8 membranous lobes, apical area of dorso-median lobe with a distinct notch, dorso-lateral lobes shorter than dorso-median lobe, ventro-lateral lobes divided into 2 lobes, ventro-median lobe short, apical area with a notch. Subgenital plate transverse and broad, posterior margin rounded. **Female.** Appearance is similar to male. Posterior margin of seventh abdominal tergite with a small process. Ovipositor narrow and long, slightly curved upwards, dorsal margin smooth, apical areas of ventral margin denticulate. Subgenital plate ligulate, apex rounded.

***Coloration*.** Body light brown. Apical area of fastigium verticis and eyes yellow. Face with 4 longitudinal black stripes. All tibia with ring-like yellowish-brown stripes, basal half of hind femur with penniform yellowish-brown stripes.

***Measurements (mm)*.** Body length: ♂23.74, ♀20.46–22.02; length of pronotum: ♂5.18, ♀5.10–5.38; length of fore femur: ♂13.60, ♀13.66–14.04; length of hind femur: ♂22.16, ♀23.42; length of hind tibia: ♂26.28, ♀26.66; length of hind basitarsus: ♂4.04, ♀4.32; length of ovipositor: 23.56–26.82.

#### Etymology.

The name of the new species refers to the ligulate subgenital plate of female. Latin “*ligul*-” referring to ligulate.

#### Distribution.

Yunnan (Maguan County).

### ﻿ Diestramima longzhouensis

Taxon classificationAnimaliaOrthopteraRhaphidophoridae

Zhu & Shi sp. nov.

547A2E05-0755-5A41-B36E-5D5DCC756CEF

https://zoobank.org/2BE2A27A-E72B-4A86-A9FD-677A920DC40E

Figs 2 , 3A, B

#### Material examined.

***Holotype***: China • ♂, Guangxi Zhuang Autonomous Region, Longzhou County, Zhubu Town, 16.VII.2021, Meng An leg. ***Paratype***: China • 1♀, Guangxi Zhuang Autonomous Region, Longzhou County, Nonggang, 22.4649°N, 106.9591°E, alt. 260 m, 16.VI.2024, Hao Xu leg.

**Figure 2.**
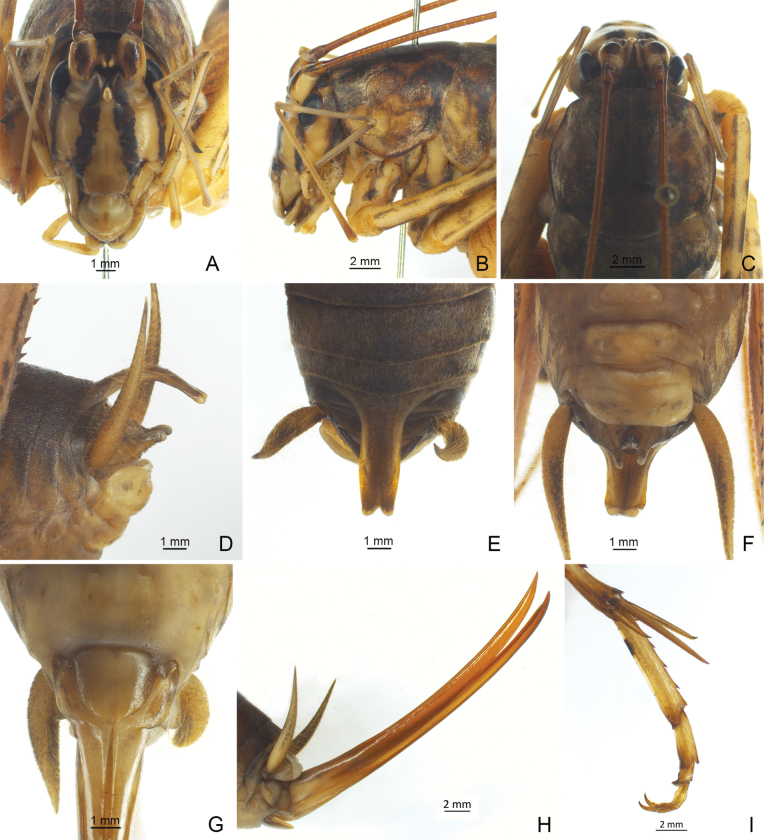
*Diestramimalongzhouensis* Zhu & Shi, sp. nov. **A–F, I** ♂ **A–C** head and pronotum **A** frontal view **B** lateral view **C** dorsal view **D–F** apex of abdomen **D** lateral view **E** dorsal view **F** ventral view **I** hind tarsus in lateral view **G, H** ♀ **G** subgenital plate **H** ovipositor in lateral view.

#### Other material.

China • 1♂, Guangxi Zhuang Autonomous Region, Dahua County, Yalong Town, Hongri Village, 23.9918°N, 107.7900°E, alt. 700 m, 20.VII.2024, Yueting Duan leg.

**Figure 3.**
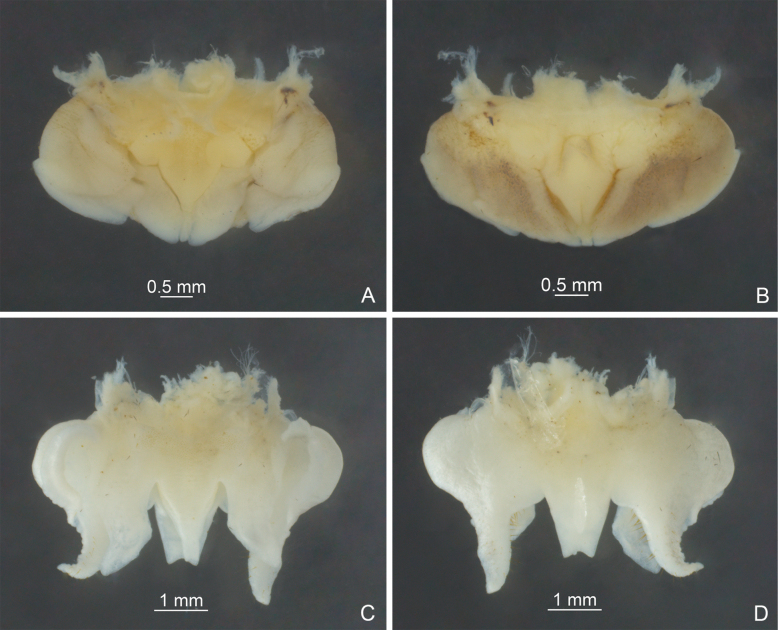
Male genitalia of *Diestramima* spp. **A, C** dorsal view **B, D** ventral view **A, B***Diestramimalongzhouensis* Zhu & Shi, sp. nov. **C, D***Diestramimanapoensis* Zhu & Shi, sp. nov.

#### Diagnosis.

The new species can be easily distinguished from congeneric known species by the shape of the seventh abdominal tergite and the paraproct of male. Posteromedian process of the seventh abdominal tergite of male long, paralleled on both sides, apical half slightly curved downwards, apical area with a notch. Male paraproct digitiform, apex obtusely rounded.

#### Description.

**Male.** Body larger than congeneric known species. Fastigium verticis with 2 conical tubercles, apices drawn together, obtusely rounded, pointing forwards. Eyes ovoid, protruding forwards; median ocellus oval, located between antennal sockets, lateral ocelli nearly circular, situated on lateral surface of basal rostral tubercles. Apical segment of maxillary palp obviously longer than subapical one, apex inflated, globular. Pronotum broad, anterior margin of disc straight, posterior margin arcuate; lateral lobe longer than high, ventral margin arc-shaped. Mesonotum and metanotum short, posterior margin of mesonotum arcuate, posterior margin of metanotum straight. Fore coxa with 1 small spine; femur unarmed on ventral surface, internal genicular lobe with 1 small spine, external genicular lobe with 1 long spine; tibia with 2 inner spines and 2 outer spines on ventral surface, apex with 1 outer spine on dorsal surface and 1 pair of spines on ventral surface, between the paired ventral spines with 1 small spine. Middle femur unarmed on ventral surface, internal and external genicular lobes with 1 long spine respectively; tibia with 2 inner spines and 2 outer spines on ventral surface, apex with 1 pair of dorsal spines and 1 pair of ventral spines, between the paired ventral spines with 1 small spine. Hind femur with 15–17 inner spines and 6–9 outer spines on ventral surface, internal genicular lobe with 1 small spine, external genicular lobe unarmed; tibia with 38 or 39 inner spines and 39–41 outer spines on dorsal surface, subapex with 1 pair of dorsal spines, apex with 1 pair of dorsal spines and 2 pairs of ventral spines, intero-dorsal spine slightly shorter than hind basitarsus; hind basitarsus with 4 dorsal spines. Posterior margin of sixth abdominal tergite straight. Posteromedian process of seventh abdominal tergite long, paralleled on both sides, apical half slightly curved downwards, apical area with a notch. Paraproct digitiform, apex obtusely rounded. Cercus narrow, conical, apex acute. Genitalia with 8 membranous lobes, apical area of dorso-median narrower than basal area, dorso-lateral lobes nearly equal to dorso-median lobe, ventro-lateral lobes divided into 2 lobes, ventro-median lobe short, apical area with a notch. Subgenital plate transverse and broad, posterior margin truncated. **Female.** Appearance is similar to male. Posterior margin of seventh abdominal tergite with a small process. Ovipositor narrow and long, slightly Curved upwards, dorsal margin smooth, apical areas of ventral margin denticulate. Subgenital plate triangular, apex rounded.

***Coloration*.** Body yellowish-brown. Face with 4 longitudinal black stripes. Fore and mid femora with ring-like black stripes, basal half of hind femur with penniform brown stripes.

***Measurements (mm)*.** Body length: ♂31.04–31.34, ♀34.92; length of pronotum: ♂8.30–8.70, ♀9.54; length of fore femur: ♂21.24–21.38, ♀22.50; length of hind femur: ♂41.04–43.82, ♀44.12; length of hind tibia: ♂47.36–48.82, ♀51.3; length of hind basitarsus: ♂8.54–8.80, ♀9.56; length of ovipositor: 30.24.

#### Etymology.

The name of the new species derives from the type locality.

#### Distribution.

Guangxi (Longzhou County, Dahua County).

### ﻿ Diestramima napoensis

Taxon classificationAnimaliaOrthopteraRhaphidophoridae

Zhu & Shi sp. nov.

2FF1E47F-6988-5A9A-9DCA-911A8BE45A12

https://zoobank.org/35C9A27B-F64C-4F5C-8B6A-590A852A4C2C

Figs 3C, D , 4 , 5

#### Material examined.

***Holotype***: China • ♂, Guangxi Zhuang Autonomous Region, Napo County, Defu village, 23.2943°N, 105.8023°E, alt. 1330 m, 13.VIII.2024, Hao Xu leg. ***Paratypes***: China • 4♂4♀, Guangxi Zhuang Autonomous Region, Napo County, Defu village, 23.2943°N, 105.8023°E, alt. 1330 m, 11–14.VIII.2024, Hao Xu leg.

**Figure 4.**
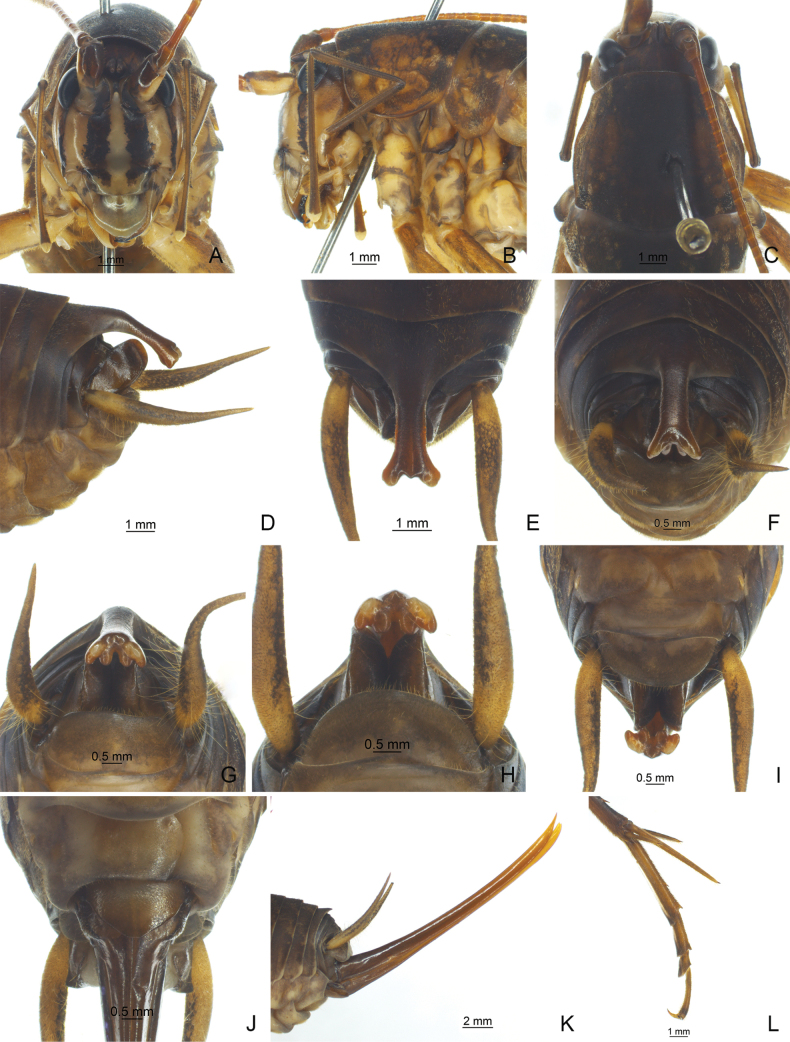
*Diestramimanapoensis* Zhu & Shi, sp. nov. **A–I, L** ♂ **A–C** head and pronotum **A** frontal view **B** lateral view **C** dorsal view **D–I** apex of abdomen **D** lateral view **E** dorsal view **F** dorso-apical view **G, H** apical view **I** ventral view **L** hind tarsus in lateral view **J, K** ♀ **J** subgenital plate **K** ovipositor in lateral view.

#### Other material.

China – Guangxi Zhuang Autonomous Region • 7♂2♀, Napo County, Defu village, 23.2943°N, 105.8023°E, alt. 1330 m, 11–14.VIII.2024, Hao Xu leg.; • 5♂6♀, Napo County, Baisheng Town, 23.0863°N, 105.5711°E, alt. 1310 m, 15.VIII.2024, Hao Xu leg.

**Figure 5.**
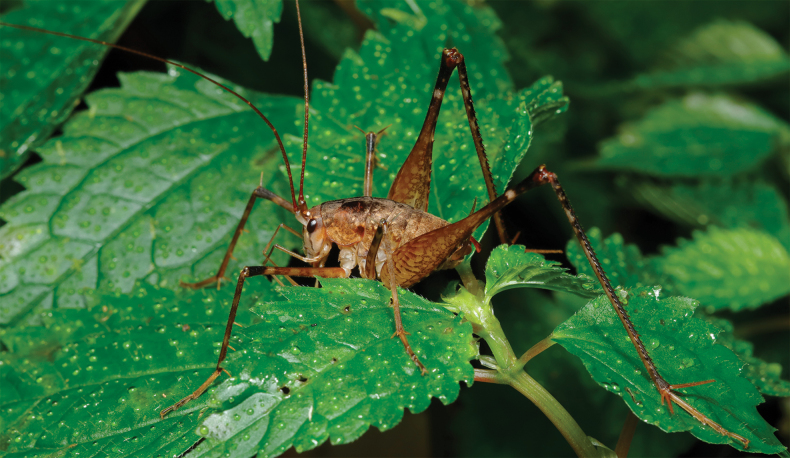
Habitats of *Diestramimanapoensis* Zhu & Shi, sp. nov. Photograph by Qianle Lu.

#### Diagnosis.

The new species is similar to *Diestramimaaustrosinensis* Gorochov, 1998, but it can be easily distinguished by the shape of the seventh abdominal tergite and the paraproct of male. Posteromedian process of the seventh abdominal tergite of male long, basal 1/3 curved backwards and downwards, apical area divided into 2 branches, each with 2 small processes. Male paraproct platelike, pointing upwards, apex rounded.

#### Description.

**Male.** Body medium-sized. Fastigium verticis with 2 conical tubercles, apices drawn together, obtusely rounded, pointing forwards. Eyes ovoid, protruding forwards; median ocellus oval, located between antennal sockets, lateral ocelli nearly circular, situated on lateral surface of basal rostral tubercles. Apical segment of maxillary palp obviously longer than subapical one, apex inflated, globular. Pronotum broad, anterior margin of disc straight, posterior margin arcuate; lateral lobe longer than high, ventral margin arc-shaped. Mesonotum and metanotum short, posterior margin of mesonotum arcuate, posterior margin of metanotum straight. Fore coxa with 1 small spine; femur unarmed on ventral surface, internal genicular lobe with 1 small spine, external genicular lobe with 1 long spine; tibia with 2 inner spines and 2 outer spines on ventral surface, apex with 1 outer spine on dorsal surface and 1 pair of spines on ventral surface, between the paired ventral spines with 1 small spine. Middle femur unarmed on ventral surface, internal and external genicular lobes with 1 long spine respectively; tibia with 2 inner spines and 2 outer spines on ventral surface, apex with 1 pair of dorsal spines and 1 pair of ventral spines, between the paired ventral spines with 1 small spine. Hind femur with 5–7 inner spines on ventral surface, internal genicular lobe with 1 small spine, external genicular lobe unarmed; tibia with 27–33 inner spines and 33 or 34 outer spines on dorsal surface, subapex with 1 pair of dorsal spines, apex with 1 pair of dorsal spines and 2 pairs of ventral spines, intero-dorsal spine slightly shorter than hind basitarsus; hind basitarsus with 2 dorsal spines. Posterior margin of sixth abdominal tergite straight. Posteromedian process of seventh abdominal tergite long, basal 1/3 curved backwards and downwards, apical area divided into 2 branches, each with 2 small processes. Paraproct platelike, pointing upwards and backwards, apex rounded. Cercus narrow, conical, apex acute. Genitalia with 8 membranous lobes, dorso-median lobe narrower, apical area with a notch, dorso-lateral lobes slightly longer than dorso-median lobe, ventro-lateral lobes divided into 2 lobes, ventro-median lobe broad, nearly equal to dorso-median lobe, apical area with a notch. Subgenital plate transverse and broad, posterior margin truncated. **Female.** Appearance is similar to male. Posterior margin of seventh abdominal tergite with a small process. Ovipositor narrow and long, slightly curved upwards, dorsal margin smooth, apical areas of ventral margin denticulate. Subgenital plate triangular, apex rounded.

***Coloration*.** Body light brown. Ocelli yellow. Face with 4 longitudinal black stripes. All femora with ring-like brown stripes, basal half of hind femur with penniform yellowish-brown stripes.

***Measurements (mm)*.** Body length: ♂19.32–22.56, ♀20.50–21.82; length of pronotum: ♂5.80–5.90, ♀5.78–6.08; length of fore femur: ♂13.80–14.72, ♀13.22–14.16; length of hind femur: ♂26.88–29.78, ♀27.34–28.04; length of hind tibia: ♂29.24–31.36, ♀28.54–30.24; length of hind basitarsus: ♂5.00–5.90, ♀5.70–5.90; length of ovipositor: 18.64–18.96.

#### Etymology.

The name of the new species derives from the type locality.

#### Distribution.

Guangxi (Napo County).

### ﻿ Diestramima parabispinosa

Taxon classificationAnimaliaOrthopteraRhaphidophoridae

Qin, Wang, Liu & Li, 2016

372937BC-3CBD-5544-AD2D-ECF01B0DF700

Figs 6 , 7A, B

Diestramima parabispinosa Qin, Wang, Liu & Li, 2016: 518.

#### Material examined.

***Holotype***: China • ♂, Guangxi Zhuang Autonomous Region, Huanjiang County, Mulun nature reserve, 18–22.VII.2015, alt. 300 m, Meiling Sun leg. ***Paratype***: 1♀, same data as for holotype.

**Figure 6.**
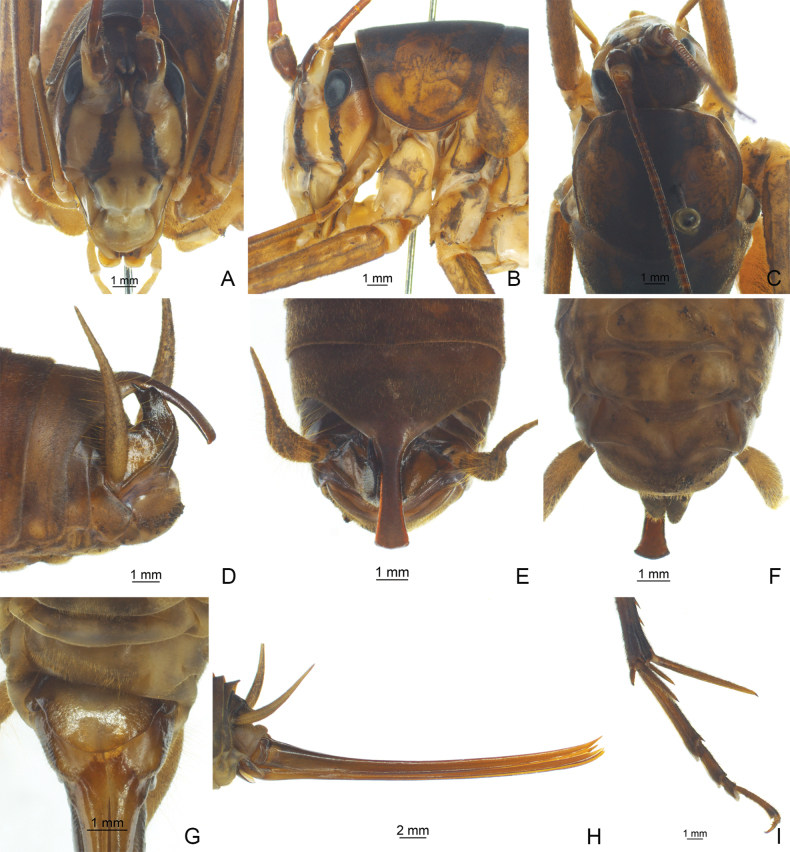
*Diestramimaparabispinosa* Qin, Wang, Liu & Li, 2016. **A–F, I** ♂ **A–C** head and pronotum **A** frontal view **B** lateral view **C** dorsal view **D–F** apex of abdomen **D** lateral view **E** dorsal view **F** ventral view **I** hind tarsus in lateral view **G, H** ♀ **G** subgenital plate **H** ovipositor in lateral view.

#### Other material.

China – Guangxi Zhuang Autonomous Region • 2♂2♀, Huanjiang County, Mulun, 25.1332°N, 107.9730°E, alt. 560 m, 28.VII.2024, Yueting Duan leg.; • 2♂1♀, Huanjiang County, Baidan Village, 25.1196°N, 108.0275°E, alt. 430 m, 4.VIII.2024, Yueting Duan leg.; • 1♂, Nandan County, Dongjia Village, 25.1131°N, 107.7472°E, alt. 840 m, 28.VII.2024, Yueting Duan leg.

**Figure 7.**
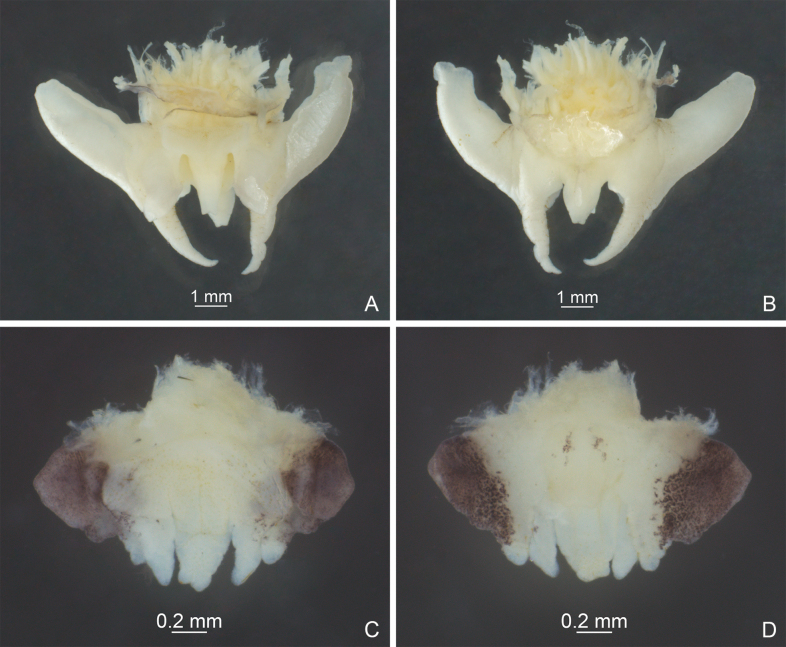
Male genitalia of *Diestramima* spp. **A, C** dorsal view **B, D** ventral view **A, B***Diestramimaparabispinosa* Qin, Wang, Liu & Li, 2016 **C, D***Diestramimadistincta* Gorochov, 2010.

#### Description.

**Female.** Appearance is similar to male. Posterior margin of seventh abdominal tergite with a small process. Ovipositor narrow and long, dorsal margin smooth, apical areas of ventral margin denticulate. Subgenital plate semi-rounded.

#### Distribution.

Guangxi (Huanjiang County, Nandan County).

#### Remarks.

The female of *Diestramimaparabispinosa* Qin, Wang, Liu & Li, 2016 is described for the first time.

### ﻿ Diestramima distincta

Taxon classificationAnimaliaOrthopteraRhaphidophoridae

Gorochov, 2010

F8F36F94-D552-5AA3-979C-0937DF9EFB50

Figs 7C, D , 8

Diestramima distincta Gorochov 2010: 14.

#### Material examined.

China • 28♂46♀, Xizang Autonomous Region, Cuona County, Senmuzha, 27.8246°N, 91.7468°E, alt. 2630 m, 15–16.VII.2024, Qidi Zhu leg.

**Figure 8.**
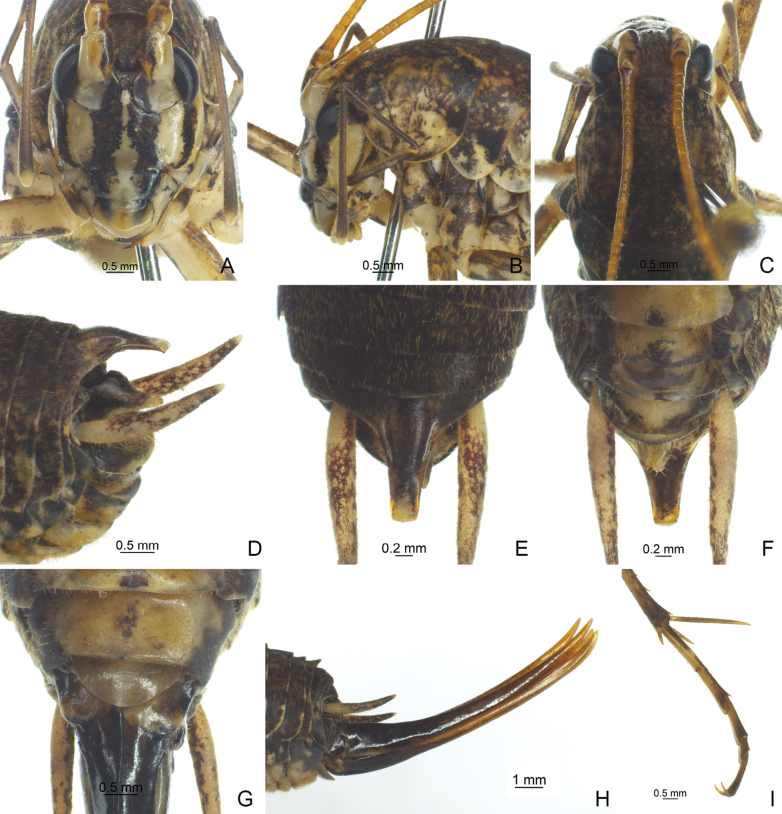
*Diestramimadistincta* Gorochov, 2010. **A–F, I** ♂ **A–C** head and pronotum **A** frontal view **B** lateral view **C** dorsal view **D–F** apex of abdomen **D** lateral view **E** dorsal view **F** ventral view **I** hind tarsus in lateral view **G, H** ♀ **G** subgenital plate **H** ovipositor in lateral view.

#### Description.

**Female.** Appearance is similar to male. Posterior margin of seventh abdominal tergite with a small process. Ovipositor curved upwards, dorsal margin smooth, apical areas of ventral margin denticulate. Subgenital plate semi-rounded.

#### Distribution.

Xizang (Cuona County).

#### Remarks.

The female of *Diestramimadistincta* Gorochov, 2010 is described for the first time.

## Supplementary Material

XML Treatment for Diestramima ligula

XML Treatment for Diestramima longzhouensis

XML Treatment for Diestramima napoensis

XML Treatment for Diestramima parabispinosa

XML Treatment for Diestramima distincta

## ﻿Additional information

### Author ORCIDs

Qidi Zhu https://orcid.org/0000-0001-8187-1286

Hao Xu https://orcid.org/0009-0004-2497-1458

Ruigang Yang https://orcid.org/0009-0009-4375-8798

Fuming Shi https://orcid.org/0000-0002-4885-9012

### Data availability

All of the data that support the findings of this study are available in the main text.
